# Supplementary material for: MetaRibo-Seq measures translation in microbiomes
Source: Nat Commun. 2020 Jun 29;11:3268. doi: 10.1038/s41467-020-17081-z (PMC7324362; doi:10.1038/s41467-020-17081-z)
Supplement: Supplementary file 10 — Supplementary Data 7 [file 41467_2020_17081_MOESM10_ESM.zip › File2/Confidence_VeryHigh_Taxonomy/91351_out.krona.html]

Javascript must be enabled to view this page.

members
magnitude
magnitudeUnassigned
count
unassigned
taxon
rank

91351\_out

5

2
5
superkingdom

phylum
5
1239

5
class
186801

5
order
186802

1
family
186803

1432051
genus
1

1
species

SRS146888\_contig\_number\_3948
1720294

541000
3
family

216851
genus
1

853
species
1

SRS147653\_contig\_number\_2674

1263
genus
2

2

SRS065504\_contig\_number\_contig-100\_68.205460SRS144183\_contig\_number\_2084
species
2293148

1898207
1

SRS020328\_contig\_number\_3810
species
